# Improved preoperative clinical staging system for intrahepatic cholangiocarcinoma using the ABC factors: a retrospective study

**DOI:** 10.3389/fonc.2026.1765119

**Published:** 2026-01-28

**Authors:** Yiyun Huang, Wenkai Ye, Junnan Huang, Linwei Xu, Yuhua Zhang, Renwei Xing, Zewei Zhang

**Affiliations:** 1 The Second School of Clinical Medicine, Zhejiang Chinese Medical University, Hangzhou, Zhejiang, China; 2 Department of Hepatopancreatobiliary Surgery, Zhejiang Cancer Hospital, Hangzhou, Zhejiang, China; 3 Hangzhou Institute of Medicine (HIM), Chinese Academy of Sciences, Hangzhou, Zhejiang, China; 4 Department of Hepatobiliary Surgery, Taizhou Municipal Hospital (Taizhou University Affiliated Municipal Hospital), School of Medicine, Taizhou University, Taizhou, Zhejiang, China

**Keywords:** ABC factor, clinical stage, intrahepatic cholangiocarcinoma, prognosis, retrospective study

## Abstract

**Objective:**

This study aimed to assess the prognostic significance of ABC factors, including anatomical factors (A), biological factors (B: carbohydrate antigen 19–9 [CA19-9]), and patient condition (C) or performance status (PS), in intrahepatic cholangiocarcinoma (ICC) patients who underwent surgery, with the ultimate goal of enhancing the preoperative clinical staging system for the diagnosis of ICC.

**Methods:**

We retrospectively analyzed the clinical data of 217 patients who underwent radical surgery for intrahepatic cholangiocarcinoma at the Zhejiang Cancer Hospital between January 2010 and August 2022. All patients were stratified into early (EA), intermediate (IM), and locally advanced (LA) stages, according to radiological findings. Multivariate Cox proportional hazards analysis was used to examine the impact of ABC factors on overall survival (OS) and disease-free survival (DFS).

**Results:**

Overall, 107 (49.3%), 86 (39.6%), and 24 (11.1%) patients were diagnosed with EA, IM, and LA, respectively. The preoperative CA19–9 level in 73 patients (33.6%) was >500 U/mL. The performance status score of 53 patients (24.4%) was ≥1. The independent risk factors for OS and DFS were IM, LA, CA19-9 >500 U/mL and performance status score ≥1. The median OS was 32.2, 20.4, 11.0, 9.8, and 6.8 months and the median DFS was 19.3, 14.8, 7.7, 5.9, and 3.3 months for patients with ABC composite scores of 0 to 4.

**Conclusion:**

ABC is an independent prognostic factor for OS and DFS in patients undergoing radical surgery for ICC. The preoperative clinical staging of patients with ICC should fully consider the anatomical, biological, and PS factors.

## Introduction

Intrahepatic cholangiocarcinoma (ICC) is the second most prevalent primary liver cancer after hepatocellular carcinoma (HCC), with a rising prevalence and incidence globally (1, 2). ICC exhibits a high degree of malignancy, with an overall 5-year survival rate of less than 8% (3). Surgery is the primary therapeutic modality for ICC; however, fewer than 40% of patients are deemed suitable candidates for surgical intervention at the time of diagnosis (4). The prognosis of patients with intrahepatic cholangiocarcinoma undergoing surgical intervention still exhibits significant variability. For surgical patients, clinical staging at diagnosis is also important to define the prognosis, guide multidisciplinary treatment, and select patients for clinical trials. Currently, the mainstream clinical staging systems are the National Comprehensive Cancer Network (NCCN) and the TNM staging system of the American Joint Commission on Cancer (AJCC) (5, 6). Tumor resectability is determined by the NCCN staging system through an assessment of the degree of vascular invasion. The AJCC TNM staging system is based on tumor size, nodal disease, and distant metastases. Neither of them carefully staged operable patients to assess their prognosis. Staging is typically more accurate after obtaining pathological results and has limited diagnostic significance.

The ABC classification was initially introduced by Katz et al. at The University of Texas MD Anderson Cancer Center (7). The classification method has been refined and elucidated in subsequent studies. It is often applied to pancreatic cancer and comprises elements A for anatomy, B for biology, and C for patient condition or performance status (PS) (8). In contrast to the AJCC staging system, the ABC classification incorporates additional factors beyond anatomical considerations to provide a more comprehensive assessment of a patient’s disease status. Anatomic factors (A) primarily pertain to the assessment of disease stage in patients using radiological imaging (9). The primary objective of biological factor (B) was to assess the preoperative levels of serum carbohydrate antigen 19-9 (CA19-9) (10). Physical fitness, activity ability, and physical consumption of the patients were assessed using PS (C) preoperatively (11, 12). The study subjects were ICC patients who had completed surgical treatment. Therefore, patients diagnosed with operable ICC were stratified into early (EA), intermediate (IM), and locally advanced (LA) stages according to radiological findings. In contrast to the World Health Organization (WHO) Performance Status score (12), the PS score in this study was modified to accommodate the limitations of retrospective analysis.

This study hypothesized that serum CA19–9 levels and modified PS scores may improve the clinical staging of ICC patients who undergo surgery. Hence, the primary objective of this study was to examine the potential prognostic significance of the ABC classification components in patients with intrahepatic cholangiocarcinoma undergoing surgical intervention. The secondary objective was to examine the collective prognostic significance of the ABC factors within a novel clinical staging framework.

## Methods

### Patients and ethics

This was a single-center, retrospective review study. All patients’ diagnoses and treatment information were obtained from Zhejiang Cancer Hospital. We retrospectively collected data from patients with ICC who underwent surgery between January 2010 and September 2022. The baseline, caregiving, diagnostic, treatment, and follow-up data of all patients were of great concern. Overall survival (OS) was the primary endpoint, and disease-free survival (DFS) was the secondary endpoint. All potential prognostic factors were assessed at baseline, ensuring that the evaluation of the OS and DFS outcome measures remained blinded. Assessors of OS and DFS were not blinded to the prognostic factors because OS and DFS are objective outcomes. Ethics statement: The study was approved by the ethics committee of Zhejiang Cancer Hospital [ethical approval number: IRB-2023-798(IIT)].

### Follow-up and research route

**Figure 1:**
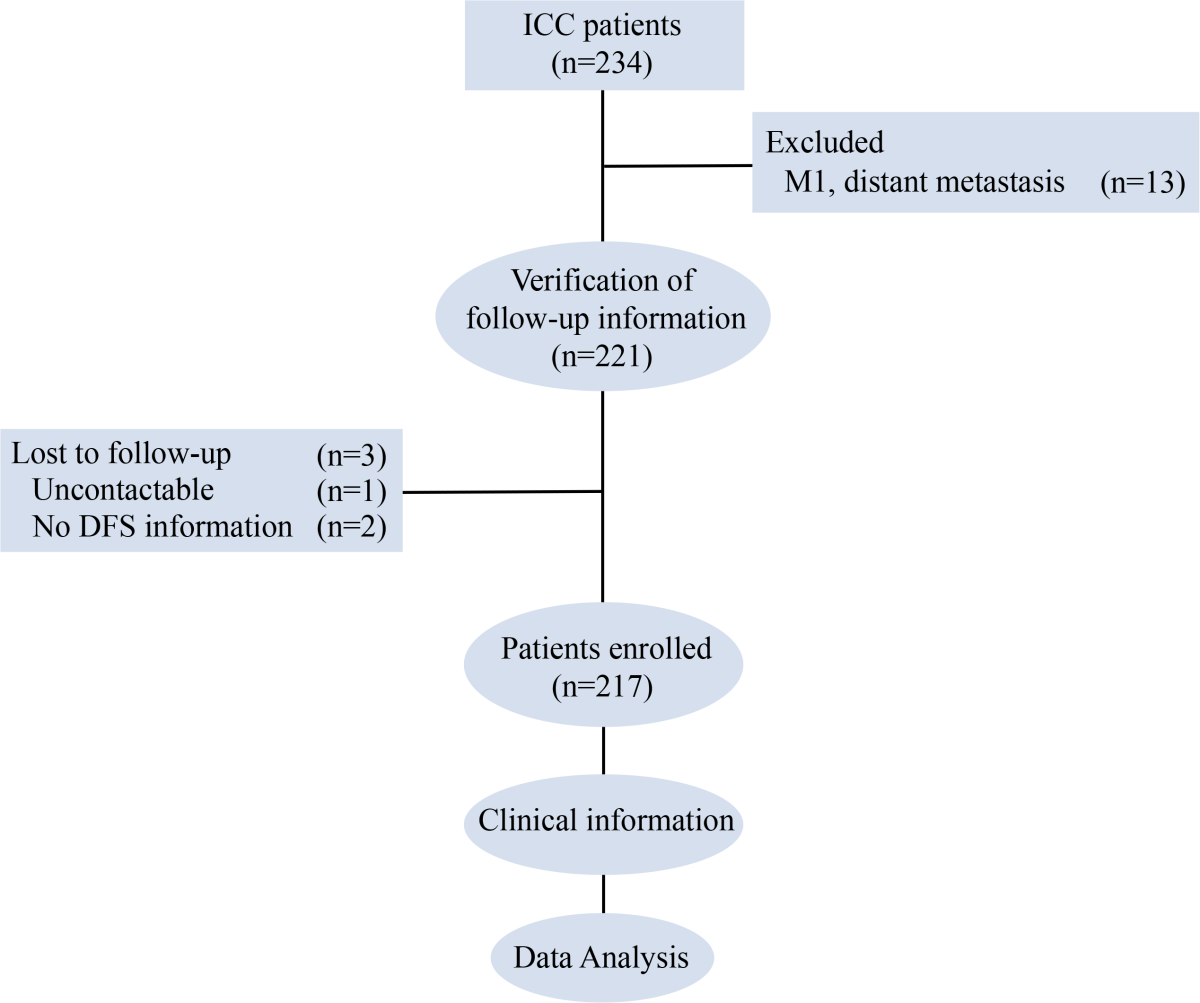
Research roadmap. ICC, intrahepatic cholangiocarcinoma; DFS, disease-free survival.

Patients undergoing surgical treatment at Zhejiang Cancer Hospital were routinely followed up. Follow-up visits were scheduled every 3 months during the first 2 postoperative years and every 6 months from years 3 to 5. Follow-up was conducted through a combination of outpatient evaluations and telephone interviews. At each visit, imaging findings (CT/MRI and contrast-enhanced CT/MRI), serum CA19–9 levels, survival status, and recurrence information were recorded. The follow-up period for this analysis ended in September 2023. A total of 234 patients were histopathologically confirmed to have ICC after surgery (**Figure 1**). Among these, 13 cases were excluded due to distant metastasis at diagnosis. Thus, 221 patients were included in the subsequent analysis. During the follow-up period, 3 patients were lost to follow-up: one could not be contacted, and two could not provide an accurate date of recurrence. Ultimately, 217 patients were included in the final analysis (**Figure 1**).

### Statistical methods

Baseline characteristics were described using frequencies and proportions for categorical variables, whereas medians with interquartile ranges (IQR) were used for continuous variables. Categorical variables were assessed using the chi-square test or Fisher’s exact probability, whereas continuous variables were analyzed using the Kruskal-Wallis rank sum test. Cox proportional hazards regression analyses were conducted to ascertain adverse prognostic indicators for OS and DFS. The selection of variables for inclusion in the multivariable analysis was based on the findings of the univariate analyses (*P* < 0.05) (13). Statistical significance was set at *P* < 0.05. CA19–9 values that fell below the lower limit of detection (i.e., CA19-9 <2 U/mL) were imputed as 2 U/mL, whereas values exceeding the upper limit of detection (i.e., CA19-9 >12000 U/mL) were imputed as 12000 U/mL.

Based on radiological findings, all ICC patients who were offered the opportunity to undergo surgical treatment were classified into EA, IM, and LA stages. The anatomical stage consisted of vascular invasion, liver capsule invasion, adjacent viscera invasion, and the estimated number of liver segments to be removed. The evaluation involving neural invasion was identified by postoperative pathological results. Neural invasion, vascular invasion, liver capsule invasion, and adjacent viscera invasion were marked as one point. The estimated number of liver segments to be removed of < 3 was marked as 1 point, and 2 points for ≥3. The radiographic tumor scores were 1–2 points for the EA stage, 3–4 points for the IM stage, and 5–6 points for the LA stage, respectively. Based on the central case data, the PS score was modified with reference to the WHO performance status[1](**Appendix Table S1**). **Supplementary Table S1** presents the detailed content of the modified PS score, while **Appendix Table S2** outlines the specific criteria of the WHO PS score. The modified PS score is primarily based on the WHO PS score. However, due to discrepancies in the data available in patients’ medical records, strict adherence to the WHO PS scoring system could result in missing values. To ensure completeness and reliability of the performance status assessment, the modified PS score was adapted according to the clinically available data in patient records. The PS score is evaluated by three independent raters using a blind method. The final result of PS score is the average value (rounded to the nearest integer). The ABC score was based on the contributions of anatomy, biology, and PS score. Participants were allocated one point for each unfavorable ABC factor and two points for LA disease.

A simplified clinical scoring system was devised by assigning points to the independent baseline prognostic risk factors for OS and DFS. Hazard ratios (HRs) were rounded to the nearest integer to determine the score for each variable. Patients with incomplete data for any ABC factor were excluded from the analysis. OS and DFS were assessed using the Kaplan-Meier method and compared using the log-rank test. Statistical significance was defined as a two-tailed P-value of less than 0.05. Statistical analyses were conducted using R version 3.5.3.

## Results

### Patients

A total of 217 patients who underwent surgical treatment for ICC were analyzed. The patient characteristics are summarized in **Table 1**. Based on radiographic imaging findings at the time of diagnosis, 107 patients (49.3%) were classified as EA, 86 (39.6%) as IM, and 24 (11.1%) as LA ICC. Based on previous data and experience, we identified 500 U/mL as the cutoff point for preoperative level of CA19-9 (14, 15). The CA19–9 level was >500 in 73 patients (33.6%). The PS score was ≥1 in 53 patients (24.4%). Surgical resection was performed with an R0 resection in 202 patients (93.1%). The median OS of all patients was 23.4 months (95% CI, 12.5 to 34.3). OS rate at 1 year was 69.2 (95% CI, 63.2 to 75.6), and 46.2% at 3 years (95% CI, 39.6 to 53.9). The median DFS of all patients was 11.3 months (95% CI, 9.1 to 13.5). DFS rate at 1 year was 48.7 (95% CI, 42.5 to 55.8), and 26.5 at 3 years (95% CI, 21.0 to 33.5).

**Table 1:** The baseline patient profiles for the ABC score.

Baseline profiles	Entire cohort (N = 217)	ABC score					*P*
		0 (n=68)	1 (n=72)	2 (n=48)	3 (n=24)	4 (n=5)	
Male sex, No. (%)	118 (54.4)	43 (63.2)	33 (45.8)	27 (56.3)	13 (54.2)	2 (40)	
Age, years, median (IQR)	60 (54-68)	60 (54-70)	64 (55-69)	60 (51-68)	60 (51-63)	55 (52-64)	0.54
Radiographic tumor stage, No. (%)							<0.001
EA (1–2 points)	107 (49.3)	68 (100)	31 (43.1)	8 (16.7)	0	0	
IM (3–4 points)	86 (39.6)	0	41 (56.9)	35 (72.9)	10 (41.7)	0	
LA (5–6 points)	24 (11.1)	0	0	5 (10.4)	14 (58.3)	5 (100)	
Baseline CA19-9, U/mL, median (IQR)	85.4 (15.8-1341.4)	19.8 (9.6-57.5)	51.6 (13.3-791.0)	1268.5 (104.4-6274.4)	1979.4 (542.8-12000)	12000 (2684.2-36141.6)	<0.001
Performance status, No. (%)							<0.001
PS 0	164 (75.6)	68 (100)	59 (81.9)	28 (58.3)	9 (37.5)	0	
PS 1	41 (18.9)	0	11 (15.3)	15 (31.3)	10 (41.7)	5 (100)	
PS 2	12 (5.5)	0	2 (2.8)	5 (10.4)	5 (20.8)	0	
BMI, kg/m^2^, median (IQR)	22.6 (20.8-25.0)	23.4 (21.7-25.3)	21.9 (20.0-24.3)	22.4 (20.8-24.9)	23.2 (20.8-25.7)	21.5 (18.2-22.0)	0.02

Imaging characteristics	Entire cohort (N = 217)	ABC score					*P*
		0 (n=68)	1 (n=72)	2 (n=48)	3 (n=24)	4 (n=5)	
Tumor size baseline, mm, median (IQR)	5.4 (4.0-7.0)	4.5 (3.8-6.0)	5.0 (4.0-7.4)	6.0 (4.0-7.9)	5.8 (4.0-7.9)	7.0 (5.8-8.0)	0.04
Number of tumors, No. (%)							0.92
Single tumor (1)	173 (79.7)	56 (82.4)	55 (76.2)	39 (81.3)	19 (79.2)	4 (80)	
Multiple tumors (≥2)	44 (20.3)	12 (17.6)	17 (23.6)	9 (18.8)	5 (20.8)	1 (20)	
Neural invasion, No. (%)	79 (36.4)	3 (4.4)	22 (30.6)	29 (60.4)	20 (83.3)	5 (100)	<0.001
Vascular invasion, No. (%)	62 (28.6)	2 (2.9)	24 (33.3)	17 (35.4)	14 (58.3)	5 (100)	<0.001
Liver capsule invasion, No. (%)	142 (65.4)	29 (42.6)	47 (65.3)	38 (79.2)	23 (95.8)	5 (100)	<0.001
Adjacent viscera invasion, No. (%)	41 (18.9)	0	8 (11.1)	16 (33.3)	16 (66.7)	1 (20)	<0.001
The expected extent of liver resection, No. (%)							<0.001

Imaging characteristics	Entire cohort (N = 217)	ABC score					*P*
		0 (n=68)	1 (n=72)	2 (n=48)	3 (n=24)	4 (n=5)	
≤3 liver segments	179 (82.5)	62 (91.2)	59 (81.9)	42 (87.5)	15 (62.5)	1 (20)	
>3 liver segments	38 (17.5)	6 (8.8)	13 (18.1)	6 (12.5)	9 (37.5)	4 (80)

Pathologic outcome	Entire cohort (N = 217)	ABC score					*P*
		0 (n=68)	1 (n=72)	2 (n=48)	3 (n=24)	4 (n=5)	
Tumor differentiation, No. (%)							0.89
Moderate/Well	58 (26.7)	18 (26.5)	19 (26.4)	14 (29.2)	5 (20.8)	2 (40)	
Poor	159 (73.3)	50 (73.5)	53 (73.6)	34 (70.8)	19 (79.2)	3 (60)	
T stage, No. (%)							<0.001
cT1	52 (24.0)	30 (44.1)	18 (25.0)	4 (8.3)	0	0	
cT2	23 (10.6)	9 (13.2)	7 (9.7)	6 (12.5)	1 (4.2)	0	
cT3	101 (46.5)	29 (42.6)	39 (54.2)	22 (45.8)	7 (29.2)	4 (80)	
cT4	41 (18.9)	0	8 (11.1)	16 (33.3)	16 (66.7)	1 (20)	
N stage, No. (%)							<0.001
cN0	157 (72.4)	61 (89.7)	53 (73.6)	28 (58.3)	14 (58.3)	1 (20)	
cN1	60 (27.6)	7 (10.3)	19 (26.4)	20 (41.7)	10 (41.7)	4 (80)	

Survival outcome	Entire cohort (N = 217)	ABC score					*P*
		0 (n=68)	1 (n=72)	2 (n=48)	3 (n=24)	4 (n=5)	
OS, months, median (95% CI)	23.4 (12.5-34.3)	51.6 (29.6-73.6)	29.5 (13.0-46.0)	11.8 (7.3-16.3)	9.6 (8.4-10.8)	6.8 (1.4-12.2)	<0.001
OS, % (95% CI)							<0.001
1-year	69.2 (63.2-75.6)	88.1 (80.7-96.2)	80.3 (71.6-90.1)	48.9 (36.5-65.5)	33.3 (18.9-58.7)	NA	
3-year	46.2 (39.6-53.9)	75.4 (65.1-87.3)	47.7 (36.8-62.0)	18.9 (9.8-36.6)	13.3 (4.0-44.9)	NA	
DFS, months, median (95% CI)	11.3 (9.1-13.5)	27.0 (15.3-38.7)	15.3 (9.1-21.5)	7.6 (6.3-8.9)	5.7 (1.7-9.7)	3.3 (1.6-5.0)	<0.001

Survival outcome	Entire cohort (N = 217)	ABC score					*P*
		0 (n=68)	1 (n=72)	2 (n=48)	3 (n=24)	4 (n=5)	
DFS, % (95% CI)							<0.001
1-year	48.7 (42.5-55.8)	74.8 (65.1-85.9)	55.6 (45.2-68.3)	25.0 (15.3-40.8)	12.5 (4.3-36.0)	NA	
3-year	26.5 (21.0-33.5)	43.3 (32.2-58.2)	33.5 (24.0-46.7)	4.8 (1.3-18.0)	8.3 (2.2-31.4)	NA	

EA, early stage; IM, intermediate stage, LA, locally advanced; CA 19-9, carbohydrate antigen 19-9; PS, performance status; OS, overall survival; DFS, disease-free survival.

### Prognostic factors for OS and DFS

Independent poor prognostic factors for OS at baseline included radiographic stage, IM (HR, 1.93 [95% CI, 1.33 to 2.81]; *P* < 0.001), LA (HR, 2.47 [95% CI, 1.38 to 4.42]; *P* = 0.002), multiple tumors (HR, 1.99 [95% CI, 1.30 to 3.05]; *P* = 0.002), CA19–9 level >500 U/mL (HR, 2.03 [95% CI, 1.41 to 3.91]; *P* < 0.001), and PS score ≥1 (HR, 2.59 [95% CI, 1.76 to 3.79]; *P* < 0.001; **Table 2**).

**Table 2:** Univariable and multivariable cox proportional hazards regression analyses of OS and DFS for patients with ICC using baseline factors.

Risk factor	Sample size	Univariable analysis (OS)		Multivariable analysis (OS)		Univariable analysis (DFS)		Multivariable analysis (DFS)	
		HR (95% CI)	*P*	HR (95% CI)	*P*	HR (95% CI)	*P*	HR (95% CI)	*P*
Sex				—	—			—	—
Male	118	1 (reference)	NA			1 (reference)	NA		
Female	99	1.30 (0.93 to 1.82)	0.13			1.29 (0.95 to 1.74)	0.11		
Age, years				—	—			—	—
<65	138	1 (reference)	NA			1 (reference)	NA		
≥65	79	1.02 (0.72 to 1.45)	0.90			0.85 (0.62 to 1.17)	0.31		
BMI, kg/m^2^									
<24	140	1 (reference)	NA	1 (reference)	NA	1 (reference)	NA	1 (reference)	NA
24-28	66	1.02 (0.71 to 1.48)	0.91	1.05 (0.72 to 1.54)	0.79	0.99 (0.71 to 1.39)	0.96	1.19 (0.84 to 1.68)	0.34
>28	11	2.31 (1.15 to 4.64)	0.02	1.82 (0.89 to 3.73)	0.10	2.19 (1.17 to 4.11)	0.02	1.63 (0.86 to 3.11)	0.14
Performance status									
PS 0	164	1 (reference)	NA	1 (reference)	NA	1 (reference)	NA	1 (reference)	NA
PS≥1	53	2.67 (1.84 to 3.87)	<0.001	2.59 (1.76 to 3.79)	<0.001	2.86 (2.03 to 4.03)	<0.001	2.87 (2.01 to 4.12)	<0.001
Radiographic tumor stage									
EA (1–2 points)	164	1 (reference)	NA	1 (reference)	NA	1 (reference)	NA	1 (reference)	NA
IM (3–4 points)	41	2.33 (1.54 to 3.52)	<0.001	1.93 (1.33 to 2.81)	<0.001	1.80 (1.29 to 2.49)	<0.001	1.62 (1.15 to 2.28)	0.006
LA (5–6 points)	12	2.31 (2.38 to 8.42)	<0.001	2.47 (1.38 to 4.42)	0.002	2.64 (1.62 to 4.27)	<0.001	2.11 (1.27 to 3.49)	0.004
Tumor size on baseline CT, mm									
0-30	21	1 (reference)	NA	1 (reference)	NA	1 (reference)	NA	1 (reference)	NA
31-60	121	1.82 (0.91 to 3.64)	0.09	1.58 (0.78 to 3.21)	0.20	1.28 (0.74 to 2.21)	0.38	1.33 (0.75 to 2.35)	0.33
>60	75	2.89 (1.42 to 5.86)	0.003	1.91 (0.92 to 3.99)	0.08	2.09 (1.19 to 3.68)	0.01	1.85 (1.02 to 3.37)	0.04
Number of tumors									
Single tumor (1)	173	1 (reference)	NA	1 (reference)	NA	1 (reference)	NA	1 (reference)	NA
Multiple tumors (≥2)	44	1.95 (1.31 to 2.90)	<0.001	1.99 (1.30 to 3.05)	0.002	1.77 (1.24 to 2.55)	0.002	1.69 (1.15 to 2.46)	0.007
Baseline CA19-9, U/mL									
≤500	144	1 (reference)	NA	1 (reference)	NA	1 (reference)	NA	1 (reference)	NA
>500	73	2.30 (1.63 to 3.24)	<0.001	2.03 (1.41 to 3.91)	<0.001	2.08 (1.52 to 2.84)	<0.001	1.99 (1.44 to 2.76)	<0.001

EA, early stage; IM, intermediate stage, LA, locally advanced; CA 19-9, carbohydrate antigen 19-9; PS, performance status.

The independent poor prognostic factors for DFS at baseline were radiographic stage, IM (HR, 1.62 [95% CI, 1.15to 2.28]; *P* = 0.006), LA (HR, 2.11 [95% CI, 1.27 to 3.49]; *P* = 0.004), multiple tumors (HR, 1.69 [95% CI, 1.15 to 2.46]; *P* = 0.007), CA19–9 level >500 U/mL (HR, 1.99 [95% CI, 1.44 to 2.76]; *P* < 0.001), and PS score ≥1 (HR, 2.87 [95% CI, 2.01 to 4.12]; *P* < 0.001; **Table 2**).

### ABC staging system

The ABC staging system diverged from the AJCC staging system by assigning 1 point to each adverse prognostic factor: anatomic (A: EA), biological (B: CA19-9 >500), and conditional (C: performance status (PS) score ≥1). It is noteworthy that the anatomic factor LA was allocated a value of two points. The patients were subsequently stratified into groups according to their cumulative scores, which ranged from 0 to 4.

**Figure 2:**
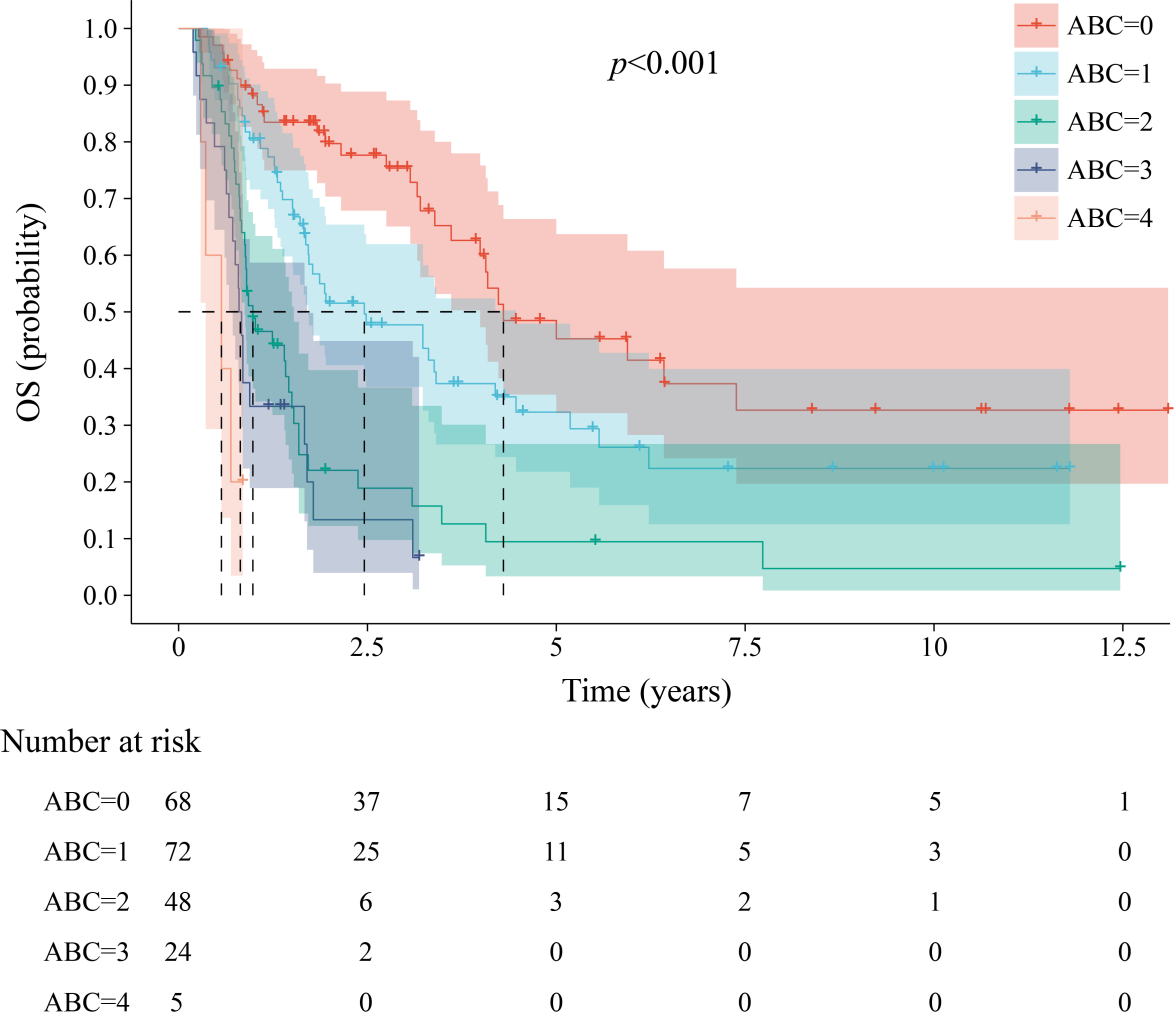
Kaplan-Meier estimates of overall survival among the groups in the ABC staging system.

**Table 1** delineates the patient and tumor characteristics along with the treatments and outcomes associated with each ABC score. The median OS for patients with scores ranging from 0 to 4 points was 51.6 months (95% CI, 29.6 to 73.6), 29.5 months (95% CI, 13.0 to 46.0), 11.8 months (95% CI, 7.3 to 16.3), 9.6 months (95% CI, 8.4 to 10.8), and 6.8 months (95% CI, 1.4 to 12.2), respectively (*P* < 0.001; **Figure 2**). The 1-year OS rates (95% CI) were 88.1% (80.7 to 96.2), 80.3% (71.6 to 90.1), 48.9% (36.5 to 65.5), 33.3% (18.9 to 58.7), and 0, respectively (*P* < 0.001). The 3-year survival rates (95% CI) were 75.4% (65.1 to 87.3), 47.7% (36.8 to 62.0), 18.9% (9.8 to 36.6), 13.3% (4.0 to 44.9), and 0, respectively (*P* < 0.001). The sample size was insufficient to reliably estimate the 5-year survival rate.

**Figure 3:**
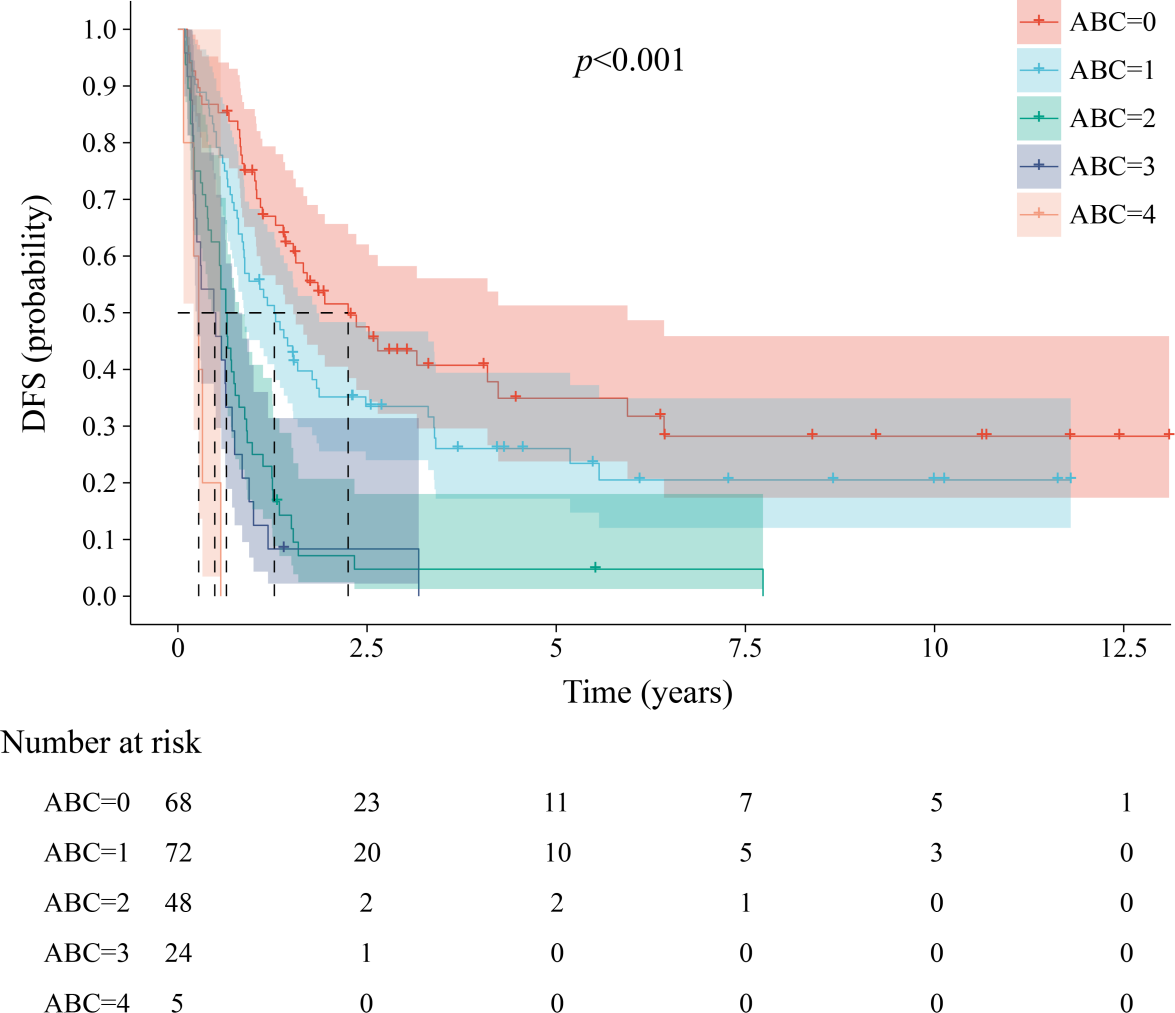
Kaplan-Meier estimates of disease-free survival among the groups in the ABC staging system.

The median DFS for patients with scores ranging from 0 to 4 points was 27.0 months (95% CI, 15.3 to 38.7), 15.3 months (95% CI, 9.1 to 21.5), 7.6 months (95% CI, 6.3 to 8.9), 5.7 months (95% CI, 1.7 to 9.7), and 3.3 months (95% CI, 1.6 to 5.0), respectively (*P* < 0.001; **Figure 3**). The 1-year DFS rates (95% CI) were 74.8% (65.1 to 85.9), 55.6% (45.2 to 68.3), 25.0% (15.3 to 40.8), 12.5% (4.3 to 36.0), and 0, respectively (*P* < 0.001). The 3-year DFS rates (95% CI) were 43.3% (32.2 to 58.2), 33.5% (24.0 to 46.7), 4.8% (1.3 to 18.0), 8.3% (2.2 to 31.4), and 0, respectively (*P* < 0.001). The sample size was insufficient to reliably estimate the 5-year disease-free survival (DFS) rate.

**Figure 4:**
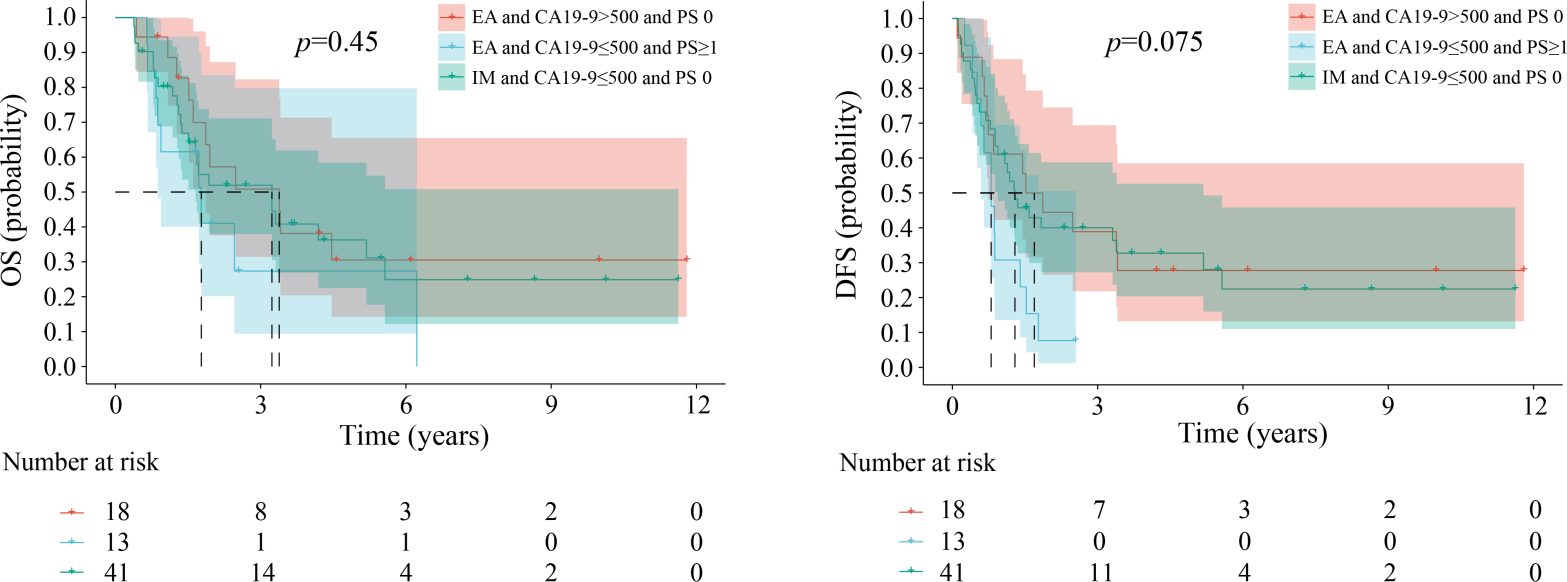
Kaplan-Meier estimates of overall survival and disease-free survival among the groups when the ABC score is 1. EA, early stage; IM, intermediate stage; LA, locally advanced; CA 19-9, carbohydrate antigen 19-9; PS, performance status; OS, overall survival; DFS, disease-free survival.

**Figure 5:**
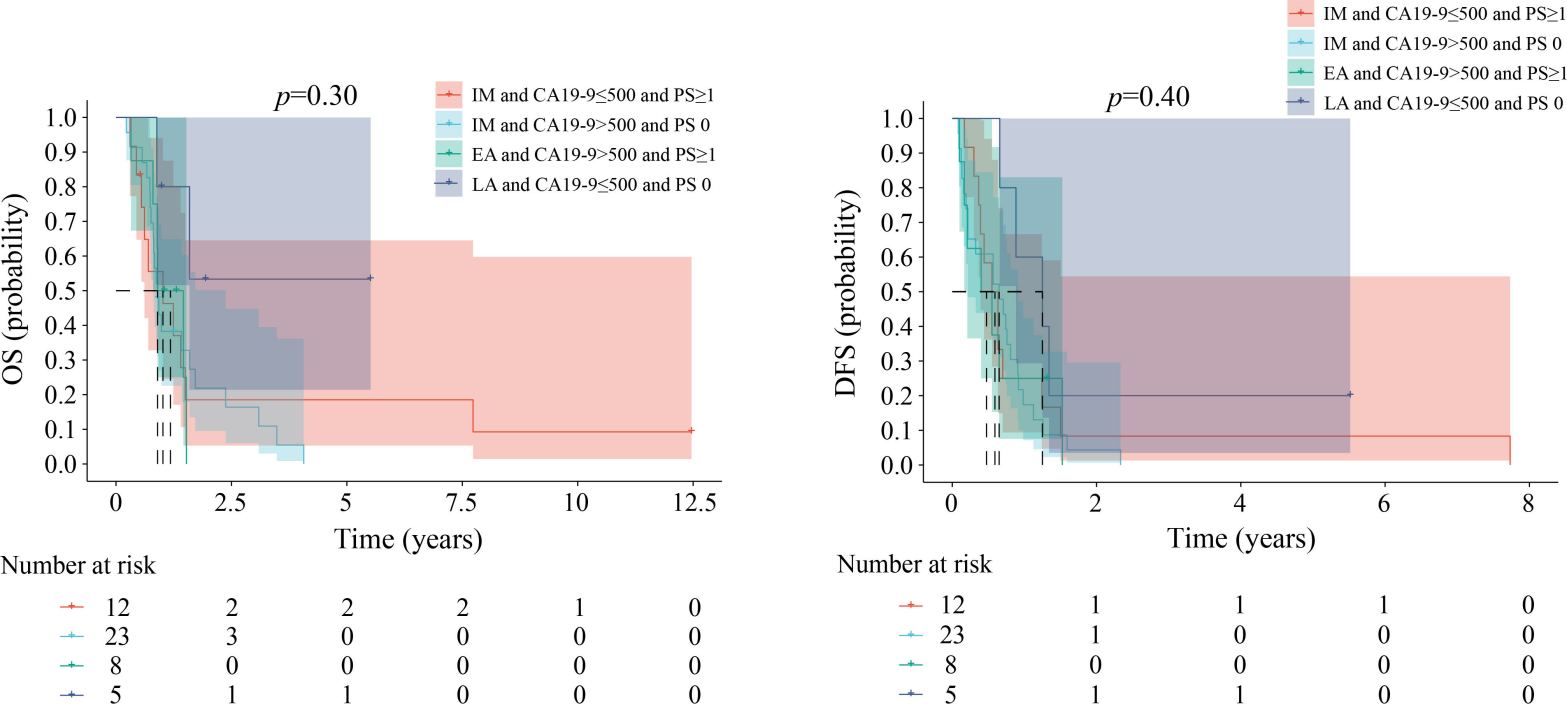
Kaplan-Meier estimates of overall survival and disease-free survival among the groups when the ABC score is 2. EA, early stage; IM, intermediate stage; LA, locally advanced; CA 19-9, carbohydrate antigen 19-9; PS, performance status; OS, overall survival; DFS, disease-free survival.

**Figure 6:**
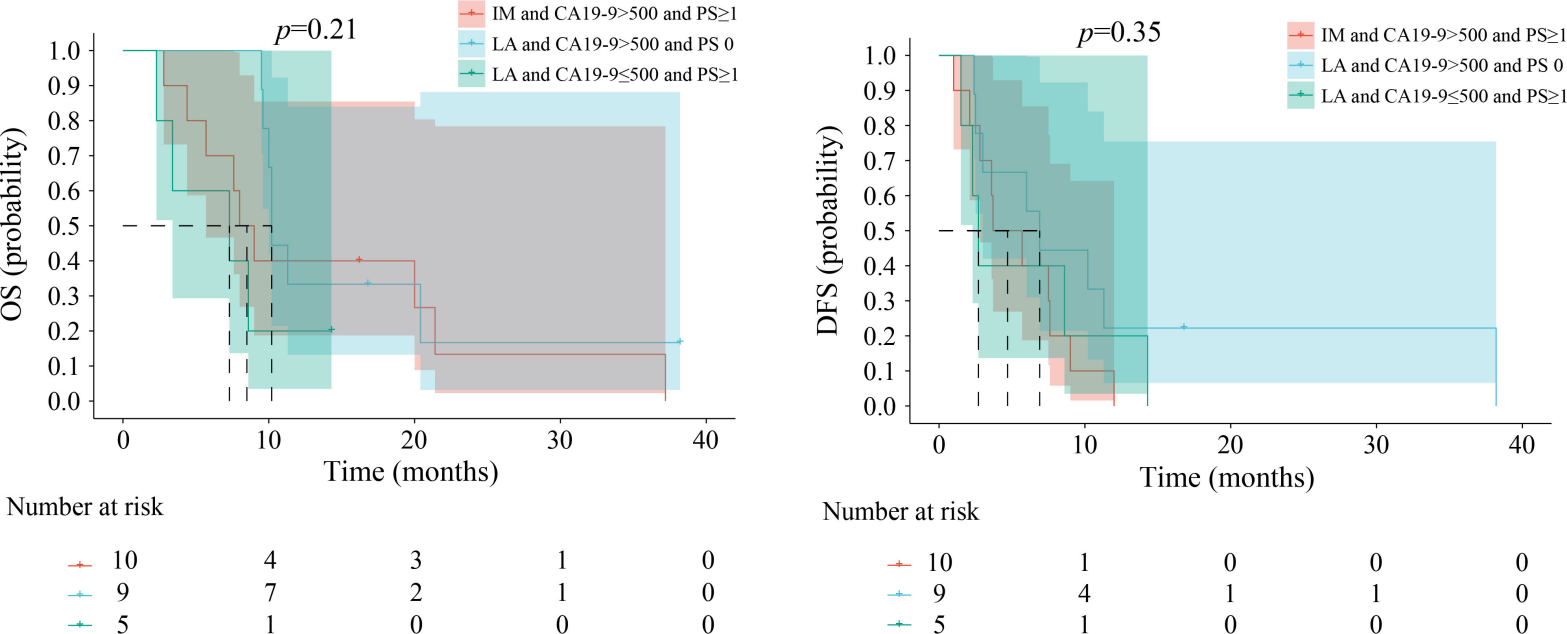
Kaplan-Meier estimates of overall survival and disease-free survival among the groups when the ABC score is 3. EA, early stage; IM, intermediate stage; LA, locally advanced; CA 19-9, carbohydrate antigen 19-9; PS, performance status; OS, overall survival; DFS, disease-free survival.

Patients with scores of 1, 2, and 3 exhibited variations in the combinations of risk factors. **Figures 4****–****6** illustrate comparable OS and DFS within each score category (1, 2, and 3). For instance, an ABC score of 1 point encompassed three distinct patient groups: those with IM ICC with CA19–9 levels ≤500 and a PS score of 0 and those with EA ICC with either CA19–9 levels >500 or a PS score ≥1. The results showed no difference in OS among the different patient groups. The DFS showed the same differences.

### AJCC/TNM staging system showed poor ability for prognosis

**Figure 7:**
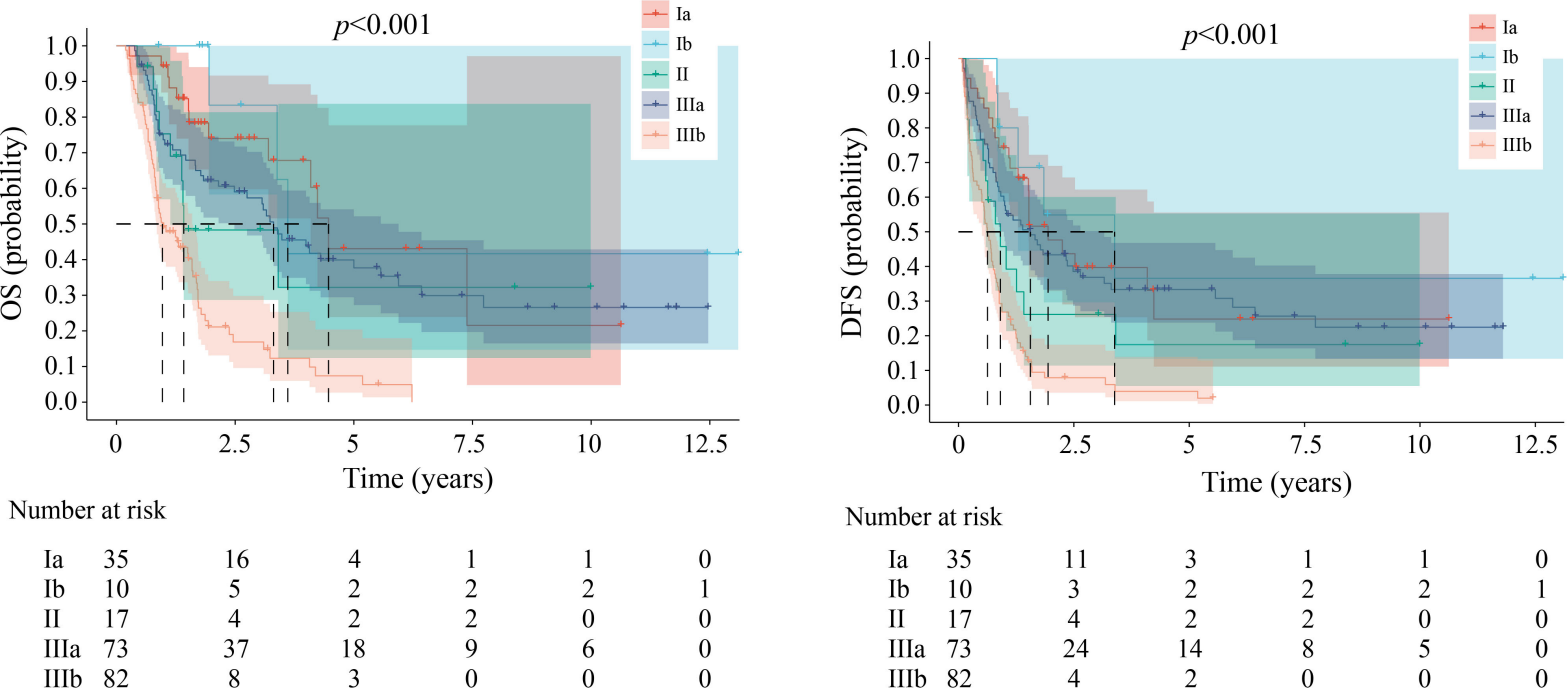
Kaplan-Meier estimates of overall survival and disease-free survival among the groups in the AJCC/TNM staging system. OS, overall survival; DFS, disease-free survival.

In this study, 217 ICC patients who underwent surgical procedures were divided into stages Ia, Ib, II, IIIa, and IIIb. The median OS for patients with AJCC/TNM stages from Ia to IIIb stages was 53.6 months, 43.3 months, 17.0 months, 39.7 months, and 11.6 months respectively (*P* < 0.001; **Figure 7**). The median DFS for patients with TNM stages from Ia to IIIb stages was 23.3 months, 40.6 months, 10.9 months, 18.7 months, and 7.6 months respectively (*P* < 0.001; **Figure 7**). Although there were significant differences in prognosis among different AJCC/TNM stages, higher AJCC/TNM stages did not predict a worse prognosis for the prognostic value. The stage IIIa had better median OS/DFS than stage II and the stage Ib had better median DFS than stage Ia with the AJCC/TNM Staging system. In this study, the AJCC/TNM Staging system showed a poor prognostic predictive ability compared to the ABC staging system. This was not an isolated event (16).

## Discussion

### Discussions on the limitations of the traditional staging system

Historically, cancer staging systems, including the NCCN staging system and AJCC/TNM cancer staging system, have predominantly relied on radiological assessments and postoperative anatomical evaluations (17). This single-center retrospective study demonstrated that the anatomic staging of operable ICC patients can be improved by evaluating two readily available clinical characteristics. Biological markers, as indicated by baseline CA19–9 levels and patient condition as assessed by performance status, were equally significant prognostic factors for operable patients with ICC. The proposed ABC staging system combines baseline assessments of anatomy (A), biology (B), and condition (C) to estimate the prognosis. The median OS for patients within the most favorable cohort was 51.6 months, whereas that for those in the least favorable cohort was 6.8 months. The median DFS for patients in the most favorable cohort was 27.0 months, while that in the least favorable cohort was 3.3 months.

Conventional TNM staging depends on tumor size, nodal status, and the presence of distant metastatic disease (18). Pathologic staging post-surgical resection is preferable to clinical staging at the time of diagnosis. Nodal status is often indeterminate during clinical staging because of the limitations of imaging techniques in accurately assessing nodal involvement (18). The likelihood of distant metastasis was substantially reduced in patients diagnosed with operable ICC. Furthermore, this study determined that the presence of multiple tumors, rather than tumor size alone, independently predicts poor prognosis for ICC, significantly complicating the TNM staging process for patients with operable ICC. Consequently, the clinical staging of patients with ICC has traditionally relied on assessing the primary tumor’s relationship with the blood vessels of the liver. During multidisciplinary team meetings, tumors are routinely evaluated based on these criteria. During multidisciplinary team meetings, tumors are usually systematically classified as resectable, borderline resectable, or advanced ICC, primarily based on the technical feasibility of margin-negative resection. However, these multidisciplinary team meetings do not apply to patients diagnosed with ICC that has been unequivocally classified as resectable. Therefore, the TNM staging system is not a certain guiding significant for guiding preoperative neoadjuvant therapies or other interventions in patients with resectable ICC.

### The advantages and applications of the ABC staging system

The integration of biological and conditional factors alongside anatomical considerations was initially employed to assess the clinical staging of pancreatic ductal carcinoma (PADC) (19). Cholangiocarcinoma is characterized primarily by the TNM staging system (20). A notable disparity persists in the long-term survival outcomes of ICC patients undergoing surgery who are classified as resectable or borderline resectable based on TNM staging (21). In this study, patients with EA/IM ICC with either poor biology (CA19-9 >500) or poor performance status (PS ≥1) were likely to have an even worse prognosis and limited benefit from surgery. Therefore, it is recommended that these patients receive neoadjuvant chemotherapy, similar to that in patients with borderline resectable cholangiocarcinoma. For such patients, neoadjuvant therapy may be recommended to reduce the ABC score, which is likely to improve patient outcomes (22). This study validated the independence of ABC factors as poor prognostic indicators for all patients diagnosed with operable ICC. The ABC staging system offers the benefits of three readily available factors for all patients upon initial presentation.

Anatomical staging has been employed to inform surgical interventions for ICC and forecast patient outcomes (23). Anatomic factors could not be accurately predicted to forecast outcomes in patients with operable ICC in our study. Tumor size was not an independent risk factor for ICC prognosis, but multiple tumors were. Several previous studies have indicated that performance status ≥2 is a significant prognostic factor for overall survival in patients with PDAC (24, 25). In this study, we found that performance status ≥1, as opposed to 0, was identified as an independent poor prognostic factor. Additionally, a performance status ≥1 was found to be significant for OS and DFS as an intermediate-staging disease ((compared with early staging) or a CA19–9 level >500. Performance status was also an independent prognostic factor.

The ABC staging system allows for the staging of all patients presenting with ICC into prognostic groups with 0–4 points. It can be used to inform patients and physicians about their prognoses. The 3-year OS rate varied from 75.4% to 0 across patients with 0–4 points. The 3-year DFS rate varied from 43.3% to 0 across patients with 0–4 points.

### The limitations of the ABC staging system

This study had several limitations. Initially, it is important to note that the composite staging system deviates from the AJCC staging system by incorporating non-anatomic factors. However, as previously elucidated, this particular system demonstrates a high level of applicability across all patients diagnosed with ICC in contrast to the AJCC system, which exhibits restricted clinical utility in individuals who do not undergo surgery (26). Second, this was a retrospective study with a potential information bias for non-surgical patients. Single-center research is also an important cause of information bias. As this study was conducted at a single center, further multicenter prospective studies are warranted to validate these findings and enhance their clinical applicability. Notably, in implementing such multicenter prospective research, standardizing imaging diagnostic criteria across participating institutions should be prioritized to minimize the influence of potential confounding factors. Third, the number of enrolled patients was too small to analyze 5-year OS with sufficient statistical power. Specifically, among patients with an ABC score of 0, the survival rate after one year was not. These patients did not benefit from surgery. Finally, anatomical disease staging surgery was conducted by different radiologists before surgery. However, despite the utilization of strict radiographic definitions, there exists the potential for inter-observer variability in the staging process, as other clinical data were not taken into consideration.

## Conclusion

In conclusion, in addition to anatomic factors (A: IM or LA), tumor biology (B: CA19-9 >500) and patient condition (C: PS score ≥1) were also important independent poor prognostic factors for patients with resectable ICC. Staging at presentation for patients with resectable ICC should be based on anatomy, CA19–9 level, and performance status. ABC is an independent prognostic factor for OS and DFS in patients undergoing radical surgery for ICC since the ABC staging system was proposed based on anatomy, CA19–9 level, and performance status. A higher preoperative ABC score indicates a poorer prognosis for the patient. It was expected to improve the prognosis by reducing the preoperative ABC score of the patients.

## Data Availability

The original contributions presented in the study are included in the article/Supplementary Material. Further inquiries can be directed to the corresponding author/s.
