# Supplementary material for: Improved preoperative clinical staging system for intrahepatic cholangiocarcinoma using the ABC factors: a retrospective study
Source: Front Oncol. 2026 Jan 28;16:1765119. doi: 10.3389/fonc.2026.1765119 (PMC12890631; doi:10.3389/fonc.2026.1765119)
Supplement: Supplementary file 1 [file Table1.docx]

Table S1. Modified performance status scores.

| Grade | Description |
| --- | --- |
| 0 | Good spirits and sleeping well, with a good appetite, and no change in weight from discomfort to diagnosis. Able to carry out all normal activities without restriction. |
| 1 | General spirits, sleeping, appetite. The weight loss < 5% from discomfort to diagnosis. Restricted in physically strenuous activity but ambulatory and able to carry out light work |
| 2 | Poor spirits, sleeping, appetite. And 5% ≤weight loss < 10% from discomfort to diagnosis. Ambulatory and capable of all self-care but unable to carry out any work activities. Up and about more than 50% of waking hours. |
| 3 | The weight loss > 10% from discomfort to diagnosis. Capable of only limited self-care, confined to bed or chair for more than 50% of waking hours |
| 4 | Completely disabled. Cannot carry on any self-care. Confined to bed or chair. |
| 5 | Dead. |

Table S2. WHO performance status.

| Grade | Description |
| --- | --- |
| 0 | Able to carry out all normal activity without restriction. |
| 1 | Restricted in physically strenuous activity but ambulatory and able to carry out light work. |
| 2 | Ambulatory and capable of all self-care but unable to carry out any work activities. Up and about more than 50% of waking hours. |
| 3 | Capable of only limited self-care, confined to bed or chair more than 50% of waking hours. |
| 4 | Completely disabled. Cannot carry on any self-care. Totally confined to bed or chair. |
| 5 | Dead. |
